# Australasian Genetic Counselors’ Perceptions of Their Role in Supporting Clients’ Behavior Change

**DOI:** 10.3390/jpm13010030

**Published:** 2022-12-23

**Authors:** Chris Jacobs, Erin Turbitt, Alison McEwen, Lou Atkins

**Affiliations:** 1 Graduate School of Health, University of Technology Sydney, Sydney, NSW 2007, Australia; 2 Centre for Behaviour Change, University College London, London WC1E 6BT, UK

**Keywords:** genetic counselors, health behavior, behavior change, behavior change techniques, COM-B model

## Abstract

Genetic testing does not always change health behavior. Effective behavior change requires a theory-driven coordinated set of activities (behavior change techniques). Genetic counselors are ideally positioned to facilitate behavior change. We aimed to explore genetic counselors’ perceptions of their role in supporting clients’ behavior change to inform the design of an intervention. Recruitment was via a professional organization and genetics services. Data were collected from 26 genetic counselors via qualitative focus groups/interview. Transcripts were analyzed using thematic analysis and mapped to the COM-B model. We identified three behaviors genetic counselors wanted clients to change: attend appointments, access information, and share information with family members. Strategies for changing clients’ behavior included: assessing needs and capabilities, providing information and support, enabling and monitoring behavior change. Barriers included lack of behavior change skills and knowledge, lack of time, and beliefs about ownership of healthcare, directiveness of behavior change, and scope of practice. Equipping genetic counselors to deliver behavior change requires (i) education in behavior change theory and behavior change techniques, (ii) integration of capability, opportunity and motivation assessment into existing practice, and (iii) development of evidence-based strategies using behavior change tools to focus discussions and promote clients’ agency to change their behavior.

## 1. Introduction

Completion of the human genome project in 2003 promised transformation of the health of society through early diagnosis, targeted treatments, and prevention of disease [1]. Raised awareness of genetic risk was expected to motivate health-related behavior change [2]. However, despite evidence that people seek genetic testing in order to make health and lifestyle changes [3,4], receiving genetic test results does not always result in behavior change [5,6,7]. The discrepancy between pre-test motivations and post-test behaviors may in part be explained by the difficulties, both for the client and the health professional, in changing health behavior [8]. 

Intervention design guidance recommends use of theory to articulate assumptions about influences on behavior [9]. Behavior change interventions are ‘coordinated sets of activities designed to change specified behavior patterns’ [10]. Behavior change techniques (BCT) are the specific components of behavior change interventions. Before a behavior can be changed, it needs to be understood in context of the behavior itself and the influences on the behavior. Through the application of theory, the influences on behavior can be identified, the interventions to change the behavior can be targeted and the appropriate behavior change techniques can be selected [11,12,13]. 

The COM-B model is increasingly applied to explore influences on health behaviors. This model proposes that for a behavior (B) to take place, there must be the physical and psychological capability (C), the social and physical opportunity (O), and the reflective and automatic motivation (M). To deliver and maintain effective behavior change, interventions must target one or more of these components. The COM-B model is mapped to the Behaviour-Change Wheel, which specifies types of behavior change interventions, enabling understanding of the target behavior in the context in which it occurs [14]. The use of behavior change techniques in combination with genetic risk information is hypothesized to result in behavior change. The extent to which behavior change techniques are currently being used in clinical genetic settings is unknown. 

Genetic counseling is a client-centered communication process that aims to help people ‘understand and adapt to the medical, psychological, and familial implications of genetic contributions to disease’ [15] (p. 79). People considering a genetic test are usually offered genetic counseling prior to testing. Currently, most pre and post-test genetic counseling is provided by specialist genetic health professionals, although as genetics becomes integrated into routine clinical care, aspects of genetic counseling will increasingly be delivered by non-genetics health professionals. Genetic counselors are accredited allied health professionals with knowledge of genetics and genomics and skills in counseling and communication, who translate complex genomic data for clients and families to facilitate understanding, informed decision making and adjustment [16]. In 2018, it was estimated that there were approximately 7000 genetic counselors worldwide, with 220 in Australasia [17]. In 2022, the number of genetic counselors working in Australasia had grown to over 400, demonstrating the rapid growth of this profession [18].

Although it has been suggested that genetic counselors are ideally placed to facilitate behavior change, the extent to which behavior change techniques are applied during genetic counseling is unclear. Our qualitative study aimed to explore Australasian genetic counselors’ perceptions of their role in supporting clients’ behavior change. Our study is the first step in developing a theory-driven intervention designed to enable the delivery of behavior change techniques during genetic counseling.

## 2. Materials and Methods

This study was reported according to the consolidated criteria for reporting qualitative research (COREQ) [19]. Ethics approval was granted by the University of Technology Sydney Research Ethics Committee (UTS HREC ETH19-4089).

### 2.1. Recruitment 

Genetic counselors working in clinical settings in Australasia were eligible to participate. We used purposive sampling to achieve two different group characteristics: those who trained 10 or more years ago to explore views of experienced genetic counselors, and those who trained less than 10 years ago to provide an indication of the skills and knowledge in behavior change gained from current training programs. We chose 10 years as the time period to define the two groups as the first Australasian master’s level genetic counseling degree was offered in 2008 [20]. Inclusion of a theory of health behavior is a curriculum requirement for Master of Genetic Counselling programs in Australasia.

We recruited participants via the email listservs of the Australasian Society for Genetic Counselors (ASGC). The invitation was circulated on three occasions. We also emailed lead genetic counselors working in public and private genetics services throughout Australasia and posted advertisements on social media. It was not possible to record the number of non-responders or the reasons for not responding. Participants were known to C.J. and A.M. and, to a lesser extent, E.T. 

### 2.2. Participants 

Twenty-nine genetic counselors agreed to participate, of which three withdrew due to busy work schedules. The 26 participants were from across Australia, with no participants from New Zealand. Participants were mostly female (*n* = 23), with 61.5% (*n* = 16) working within a clinical genetics department. The total mean number of years since completing training was 10.5 (SD = 7.0). The mean number of years for those <10 years since graduating was 7 (SD = 5.6). The mean number of years for those >10 years since graduating was 13.6 (SD = 6.5)

Some behavior change training was reported by 19.2% (*n* = 5) of participants (participant characteristics are shown in Table 1). To protect confidentiality amongst this small sample population, race and ethnicity data were not collected and quotations were allocated to focus groups rather than individual participants. 

### 2.3. Procedure

Participants completed a demographic survey and had the option of attending a virtual semi-structured focus group or one to one interview. 

The topic guide was informed by our knowledge and experience of genetic counseling and behavior change research. Four key areas were addressed: what genetic counselors want clients to do after genetic counseling; influences on client behavior; health behavior change strategies delivered by genetic counsellors; and influences on genetic counselors’ behavior in delivering these strategies. We piloted the topic guide with three clinical and academic genetic counselors in a pilot focus group. The pilot indicated some minor changes to the topic guide, for example, development and inclusion of an example behavior was suggested, in case the focus group participants were unable to think of any behaviors.

We conducted five semi-structured focus groups and one interview via Zoom between February 2020 and July 2021. The focus groups were facilitated by ET with CJ present as a note taker. The interview was conducted by CJ. We informed participants of the purpose of the study and the researchers’ qualifications, genetics background and interest in the study. For the focus groups, we used Krueger’s format whereby a ‘round robin’ open question was asked to engage all participants, followed by transition questions, and ending with focused closing questions [20]. The focus groups lasted on average 80 min (range 72–93 min) and the interview lasted 40 min. 

### 2.4. Data Analysis

Using deductive inductive qualitative content analysis [21], CJ and ET deductively coded the transcripts in NVivo according to the COM-B model. We then coded similar responses to the same components of the COM-B model [10]. In discussion with the research team, we inductively generated labels to describe the themes and organized these data using a logic model, including client behaviors, influences on client behaviors (COM), genetic counseling strategies, and influences on genetic counselors’ behavior (COM). 

## 3. Results

### 3.1. Behaviors Genetic Counselors Wanted Clients to Change following Genetic Counseling

We identified three clusters of desired behavioral outcomes of genetic counseling:

- Attend recommended screening/health appointments and change health behavior. Examples of this behavior were: being vigilant about attending breast surveillance, reducing screening if not required, taking folic acid in preparation for a pregnancy and quitting smoking.
- Access information about the condition and management and communicate information to other agencies. Examples of this behavior were: re-contacting the genetics service and other reliable agencies, knowing where and how to access support services, and talking knowledgeably and confidently about own and family’s genetic information with, for example, partners, health professionals and teachers.
- Share accurate information with relevant family members. Examples of this behavior were: knowing what and how to disseminate risk and genetic testing information and sharing the information with relevant family members.

### 3.2. Strategies Used by Genetic Counselors to Help Clients Achieve These Behaviors and the Influences on Genetic Counselors’ Behavior

We identified four clusters of strategies used by genetic counselors to achieve these behaviors: assess needs and capabilities, provide tailored information, resources and support, enable behavior change, and monitor behavior change. These strategies and the influences on genetic counselors’ behavior in delivering them are detailed below. (Full quotes are shown in Supplementary Table S1.)

#### 3.2.1. Assessing Needs and Capabilities 

Genetic counselors’ strategies for assessing clients’ capability to attend appointments and share information with relatives included careful listening and questioning to establish support networks, asking questions, identify coping styles, and establish plans. In assessing the likelihood that clients will change their behavior, participants described drawing on intuition—‘*I guess some of it is you kind of eyeball the person and get a good hunch as to whether they are going to follow up or not’* (FG4). 

The influences on genetic counselors’ behavior to deliver strategies for assessing needs and capabilities are detailed below:

- Capability: Participants identified genetic counselors’ skills at working with families. They also described how genetic counselors are able to develop rapport with clients and families and the tendency to ‘*support one person over the other based on the rapport’* they had developed during the consultation (FG2).
- Motivation: Beliefs about the extent to which clients should take ownership of their own healthcare was identified as a motivation influencing genetic counselors’ behavior. Participants commented that the ‘*responsibility and ownership*’ (FG3) of healthcare belonged to the client and that clients need to want to ‘*help themselves*’ (FG4). A further motivator influencing genetic counselors’ behavior was judgements of clients’ ability to manage their own healthcare. Participants spoke about how their decision to provide follow up, for example to monitor clients’ attendance at appointments, was influenced by their judgement of the clients’ vulnerability or capability to do so.

#### 3.2.2. Providing Tailored Information, Resources and Support

Participants described providing information about topics such as the genetic condition, genetic testing, risk management, screening, who to connect with and how to access support groups. Genetic counselors translate, tailor and filter complex genetic information to help clients understand and apply the information in the context of their own and their family circumstances, for example attending health appointments. The focus is on *‘what they [*clients*] perceive as important to them. It is all about tailoring the information to their needs’* (FG2). This strategy was designed to help clients ‘*filter that information online in the future if they do need to find something else’* (FG3). Genetic counselors use a variety of tools to deliver information, including post-consultation summary letters, information sheets and follow up emails. Participants explained that to enhance understanding, they often provide accessible and relevant information verbally and in writing. 

Strategies for providing access to relevant support and resources, included ‘*implementing*’ (FG4), ‘*facilitating*’ (FG1) and ‘*linking*’ (FG2) clients to professional or lay individuals or networks and communities of people with similar experiences. 

The influences on genetic counselors’ behavior to deliver strategies for providing tailored information, support and resources are detailed below:

- Capability: Participants highlighted that genetic counselor training equips them with the skills to understand, filter and convey relevant information and address misconceptions, ‘*One of the things that genetic counselors do really well—is that translation of really complex genetic information*’ (FG1). They talked about the challenges of helping people to understand the significance of genetic information. Some noted a rise within the last five years in the number of clients who are ‘*over-literate’* or ‘*mis-literate’* (FG1) and who attend clinic with information, terminology and expectations sourced from the internet, not all of which is reliable or relevant and much of which they do not understand, and which the genetic counselor needs to ‘*disprove*’ (FG1).
- Opportunity: Genetic counselors’ wealth of local knowledge and connections enables them to help clients access the health appointments, social services and support that they need, for example knowledge of local transport or ‘*how to get a family in touch with the fuel rebate so they can get themselves to appointments*‘ (FG3). The connectedness of genetic counselors with local agencies also influenced their ability to help with clients’ access and, when working with families from diverse cultural groups, drawing on key family knowledge and knowing how to contact services.
- Motivation: There was a belief that the professional role of genetic counselors involves coordinating all aspects of care for clients and families with genetic conditions, including facilitating access to information, support and resources. Participants noted that the genetic counselors’ role includes a ‘*huge spectrum of responsibility’* ranging from providing practical information, helping navigate the healthcare system and the transport system and helping people to think about how the information will affect their *‘existence in the world’* and the way they assimilate it (FG3).

#### 3.2.3. Enabling Behavior Change 

To enable behavior change, participants spoke about promoting collaborative decision-making and supporting planning. Genetic counselors also activate health and screening appointments. 

A further strategy identified for enabling behavior change was encouraging self-management and behavior change by ‘*providing time’* and ‘*exploring … barriers in a non-directive way’* (FG2), gently challenging priorities and negotiating about what the genetic counselor and client will do to ensure clients change their behavior. One participant explained, ‘*I might say “I am going to do A, B, C, why don’t you tell me a little bit about what you are going to do after you see me?”. Engaging them in a session, allowing them to think for themselves what they need to do’* (FG4).

Participants described several strategies for enabling the sharing of information with family members and other agencies. Facilitating family communication involved helping clients identify and plan which relatives to share information with; what information to share with relatives; negotiating what can be said to whom and how the information will be shared. In sensitive or difficult family relationships, further strategies were explained such as incorporating the ‘*genetic story at an age-appropriate stage*’ (FG1) or imagining ‘*how they could make contact’* with estranged relatives (FG1). To facilitate the sharing of information within families, participants provided skills and resources such as helping clients to rehearse conversations, providing an anonymous family letter, using skills like teach back to assess understanding and role plays. An example given for how genetic counselors equip clients to share information with other agencies was providing a ‘*general information letter that someone can take to [their child’s] teachers* (FG3). 

The influences on genetic counselors’ behavior to deliver strategies for enabling behavior change are detailed below:

- Capability: Genetic counselors’ training in the prevention and early detection of relatives’ risk was considered a facilitator in enabling behavior change: *‘We are quite good at helping patients with that, within our scope of practice* (FG2)’. However, the lack of skills and training in behavior change techniques and a resulting lack of confidence to deliver behavior change was perceived as a barrier along with a lack the willingness or capacity amongst some genetic counselors to learn new skills.
- Motivation: Beliefs about changing clients’ behavior, non-directiveness and genetic counselors’ role motivated the genetic counselors’ behavior. Participants expressed beliefs about the directiveness of behavior change. They worried about being ‘*too pushy*’ (FG2, FG5) and questioned whether the ethos of non-directiveness was *‘a little bit at odds with behavior change’* (FG2). Contrasting views were expressed about whether genetic counseling should be directive or non-directive, for example, ‘*you want to be non-directive as you don’t want to push people to say, go and have risk-reducing surgery’* (FG2) and *‘[non-directiveness] doesn’t actually align with what we do’* (FG5). Participants spoke about how *‘non-directiveness is from before, and now, with the mainstreaming we have to be much more directive*’ (FG5). There was also a belief that whilst behavior change is ‘*an integral part* ‘of genetic counselors’ work (interview 1) and there is a ‘*moral responsibility’* to support behavior change (FG5), it may not be the role or responsibility of genetic counselors to deliver behavior change.

#### 3.2.4. Monitoring Behavior Change

Participants spoke about monitoring clients’ attendance at appointments and their health behavior change through regular follow up, for example checking that people at high risk of cancer have attended their annual screening appointments or had their recommended blood tests. One participant described having briefly piloted a tool for monitoring the outcome of family communication. 

The influences on genetic counselors’ behavior to deliver strategies for monitoring behavior change are detailed below:

- Opportunity: Genetic counselors’ ‘*limited time’* and ‘*giant workload’* (FG3), lack of available resources for some genetics services and the limited number of appointments with each client were identified as barriers to monitoring clients’ attendance at appointments or the outcome of family communication. Alongside this, limitations in the system for organising appointments, such as lack of access or long waiting times, and the lack of ‘*structural follow up’* (FG4), were also considered to be barriers to monitoring clients’ behavior change.

A summary of the enablers and barriers influencing genetic counselors’ capability, opportunity and motivation to deliver behavior change and an outline of what genetic counselors need to know and do to deliver behavior change is shown in Table 2.

## 4. Discussion

Following genetic counseling, participants wanted clients to attend recommended health appointments, access information and management and communicate with other agencies, and share accurate information with relevant family members. The genetic counseling strategies employed in current practice to achieve these client behaviors involved assessing clients’ needs and capabilities, providing tailored information, resources and support, and enabling and monitoring behavior change. Genetic counselors have several of the capabilities required to change behavior, including skills in working with families, establishing rapport, and understanding, filtering and conveying relevant information. However, their opportunities and motivations to deliver behavior change are more limited. Barriers include lack of skills and training in behavior change, limited time, capacity and resources, and beliefs about clients’ capability, directiveness of behavior change, and genetic counselors’ scope of practice. Enabling genetic counsellors to deliver behavior change requires knowledge of how to apply behavior change theory to assess capability, opportunity and motivation and the strategies to use to change clients’ behavior, and development of strategies using behavior change tools to focus discussions and promote clients’ agency to change their behavior.

The client behavioral outcomes we identified fall within the remit of the genetic counselors’ role which includes facilitating understanding, informed decision-making and adjustment [16]. The widely recognized definition of genetic counseling and the genetic counseling scope of practice documents of the British and Australasian genetic counseling professional bodies all specify helping clients to adapt or adjust [15,22,23]. A systematic review of the role of the genetic counselor noted that, the empathic client-centered approach upon which genetic counseling is based, enables and invites emotional expression thereby facilitating adaptation and supporting effective decision making [24]. The Oxford English Dictionary provides a definition of the verb to adapt as ‘To become adjusted or used to new conditions; to change one’s behavior or attitude to suit a different environment’ [25]. 

In the face of a new genetic diagnosis, people may need to make long term behavior changes that will in some way have a positive impact on the future genetic health and wellbeing of themselves and their families. Facilitating behavior change, may therefore be a key factor in facilitating adaptation to a genetic condition. To effectively change health behavior is challenging for clients and health professionals and requires an understanding of the client’s capability, opportunity and motivation to change as well as application of behavior change techniques. Awareness of the behaviors that genetic counselors want clients to change is helpful in targeting the strategies to use to change those behaviors. 

We found that genetic counselors do utilize some strategies to help clients to change the desired behaviors, however these strategies may not be intentionally focused on behavior change. Some of the difficulty for genetic counselors in identifying what they do to change clients’ behavior lies in the challenge of defining what is meant by behavior. Behavior can be defined as “Anything a person does in response to internal or external events. Actions may be overt (motor or verbal) and directly measurable or, covert (activities not viewable but involving voluntary muscles) and indirectly measurable; behaviors are physical events that occur in the body and are controlled by the brain” [26] (p. 327). This definition differentiates behavior, which is a physical event or outcome, from decision-making which is a cognitive process involving emotions and cognitions that influence behavior [27]. The strategies we identified were largely focused on assessing clients’ information and support needs, rather than assessing their capability, opportunity or motivation to change their behavior [28]. Several studies have found that genetic counselors tend to prioritize didactic provision of biomedical information over psychotherapeutic counseling [28]. Although there is some evidence that explanation of the physiological processes underlying disease risk and taking protective action can promote beliefs that motivate protective behavior, provision of targeted information alone does not necessarily or always result in behavior change [5,6,7]. Our participants did identify examples of enabling behavior change, mostly in relation to sharing genetic information within families. Monitoring the outcome of any behavior change strategies was however limited to the ad hoc provision of follow up. For the most part, what the participants referred to as behavior change strategies were not focused on changing physical events or outcomes but rather on the emotions and cognitions that influence behavior. This is unsurprising given that few of our participants had received any training in behavior change.

Our participants described genetic counselors’ ability to work with families and develop rapport. Although assessment of risk, understanding, psychological and support needs are all part of the genetic counselors’ role, assessing clients’ capability, opportunity and motivation to change behavior does not currently fall into the remit of genetic counselors. Participants described genetic counselors’ capability to communicate tailored, accessible information about genetic risk and management to clients. There was evidence of capability to enable behavior change for the three identified client behaviors, most notably regarding facilitating family communication about genetic information. However, there is no evidence of training for genetic counselors in assessment of clients’ capability, opportunity and motivation to change behavior, knowledge and skills to motivate clients to change their behavior, or skills and training in the delivery of behavior change techniques. To address the gaps in genetic counselors’ capability requires knowledge of behavior change theory, how to apply the theory and which behavior change techniques and strategies are effective.

Genetic counselors expect to assess clients’ family history, risk, understanding, psychological and support needs. It is not however within their expectations to assess clients’ capability, opportunity and motivation to change behavior. Participants reported that genetic counselors had good local knowledge and multiple connections, enabling them to provide information about local services, resources and support. However, the opportunities for monitoring clients’ behavior change after the genetic counseling appointment were limited by lack of time, capacity and resources and inconsistent and under-funded health systems. To harness the opportunity for genetic counselors to deliver behavior change strategies requires efficient modification of existing practice, rather than adding to the workload. For example, genetic counsellors might be able to make a quick behavioral diagnosis by asking questions about capability, opportunity and motivation to change behavior as part of the assessment of family history and support. The PRIMROSE study describes a parallel example whereby busy practice nurses were able to introduce a behavior change intervention into their practice aiming to reduce cholesterol and cardiovascular disease risk in people with severe mental illnesses [29]. This study found that similar barriers and facilitators existed for practice nurses implementing behavior change techniques to those identified by our study [30].

We found a belief that clients should take ownership of their own health, and that the role of the genetic counselor includes acting as a care coordinator. Alongside this we identified judgements about clients’ lack of ability to manage their own healthcare. Participants were motivated to promote collaborative decision-making and follow up clients after genetic counseling. We found mixed beliefs about whether behavior change is directive or not, whether genetic counseling should be directive or non-directive and whether behavior change is within their scope of practice. These beliefs arguably reflect ongoing debate about the role of non-directiveness in genetic counseling [31]. To address the gap in genetic counselors’ motivation to deliver behavior change, further research is needed to identify the strategies that will enable genetic counselors to use the COM-B model collaboratively with clients. Such strategies will need to help focus the discussion on behavior change, help clients believe that change is possible and enable clients to have agency to change their behavior. 

## 5. Limitations and Future Research

This study drew on expertise in genetic counseling, behavior change and qualitative research, and used a systematic, evidence-based theoretical framework (the COM-B model) to explore the barriers and facilitators to genetic counselors’ delivery of behavior change techniques. However, the exploratory nature of the study meant that the genetic counseling strategies and the influences on genetic counselors’ behavior were not always linked by the participants to the behaviors genetic counselors wanted clients to change. In addition, some of the influences on genetic counselors’ behaviors may apply to several of the genetic counseling strategies and client behaviors. Perhaps because genetic counselors are more familiar with emotion than behavior, the participants found it difficult to identify specific behaviors and behavior change strategies. To draw out meaningful findings from the data therefore required interpretation by the research team. It should be noted that, as with all qualitative research, our findings are based on participants’ perceptions of environmental influences. Replication of this study with different populations and settings and triangulation using quantitative methods, including audit of workloads and budgets, would help to validate these findings. 

Having gathered views and experiences of Australasian genetic counselors about their current practice in helping clients to change their behavior, the next phase of designing a behavioral intervention [14] is to establish what is required to help genetic counselors deliver behavior change. This research may include a systematic review of the behavioral interventions used in genetic counseling, alongside gathering the views of genetic counselors, clients and behavior change experts about what would be helpful. These data will be used to produce a prototype intervention which will then need to be implemented and evaluated for validity, acceptability, and evidence that the intervention changes outcomes.

## 6. Conclusions

Bridging the gap between clients’ pre-test intentions and post-test behaviors requires behavioral intervention from genetic health professionals. Genetic counselors already have some of the capabilities required to deliver behavior change techniques. However, to effectively deliver behavior change, genetic counselors need knowledge of behavior change theory, skills to integrate assessment of capability, opportunity and motivation into existing practice and evidence-based behavior change strategies to focus discussions and promote clients’ agency to change their behavior.

## 7. Research Team

C.J. (female) is a UK Registered Genetic Counselor, Registered Nurse, and senior lecturer in Genetic Counseling at UTS. E.T. (female) is a social scientist and senior lecturer in Genetic Counseling at UTS. A.M. (female) is an Australasian registered genetic counselor, associate professor, and former head of the discipline of Genetic Counseling at UTS. C.J., E.T. and A.M. have qualitative research experience. L.A. (female) is a a researcher, trainer and consultant in behavior change intervention design and evaluation. L.A. is senior teaching fellow of the University College London (UCL) Centre for Behaviour Change and leads the Australasian Hub. L.A. and E.T are not members of the ASGC although C.J and A.M. are members.

## Figures and Tables

**Table 1:** Participant characteristics.

Characteristic	%	*n*
*Gender* Female Male	88.5 11.5	23 3
*State* VIC NSW WA QLD TAS	34.6 26.9 23.1 11.5 3.8	9 7 6 3 1
*Behavior change training* Yes No	19.2 80.8	5 21
*Work context* Genetics dept Subspecialty dept Other	61.5 7.7 30.8	16 2 8
*Specialty* Cancer General Reproductive Cardiac Pediatrics Research	38.5 26.9 19.2 7.7 3.8 3.8	10 7 5 2 1 1

**Table 2:** Enablers and barriers to genetic counselors’ ability to deliver behavior change and what genetic counselors need to know and do to deliver behavior change.

COM-B Components	Influences on Genetic Counselors’ Ability to Deliver Behavior Change		What Genetic Counselors Need to Know/Do to Deliver Behavior Change
	Enablers	Barriers	
Psychological capability	Skills in: - working with families - developing rapport - understanding, filtering and conveying relevant information and addressing misconceptions - prevention and early detection of relatives’ risk	Lack of knowledge and skills to: - assess capability, opportunity and motivation to change behavior - motivate clients to change their behavior - deliver behavior change techniques	Knowledge of how to apply behavior change theory and which behavior change techniques and strategies are effective.
Opportunity	- Assessment of family history, psychological and support needs and understanding of genetics - Access to local knowledge and connections	Lack of: - time, capacity and resources to follow up - consistent/reliable follow up systems	Modification of existing practice to incorporate questions about capability, opportunity and motivation to change behavior into existing assessment
Reflective motivation	- Belief that clients should take ownership of their own health - Belief in role as care coordinator - Promote collaborative decision-making and follow up	- Belief that some clients lack ability to manage own healthcare - Mixed beliefs about directiveness/nondirectiveness of behavior change, genetic counseling, and scope of practice	Strategies to focus discussion with clients about behavior change, and promote agency to change behavior

## Data Availability

The data presented in this study are available on request from the corresponding author. The data are not publicly available to maintain the privacy of participants.

## Supplementary Materials

The following supporting information can be downloaded at: https://www.mdpi.com/article/10.3390/jpm13010030/s1, Table S1: Full quotes.
