# Supplementary material for: Australasian Genetic Counselors’ Perceptions of Their Role in Supporting Clients’ Behavior Change
Source: J Pers Med. 2022 Dec 23;13(1):30. doi: 10.3390/jpm13010030 (PMC9863991; doi:10.3390/jpm13010030)
Supplement: Supplementary file 1 [file jpm-13-00030-s001.zip › jpm-2070640-supplementary.pdf]

Table S1. Full quotes

Genetic counselors' strategies	Influences on genetic counselors' behavior
**Assess needs and capabilities:**<br>Listening and questioning<br>– *It is about management of that condition and adjustment to that condition. I guess what supports do they need in place to adjust to that diagnosis?* (FG4)<br>– *Kind of working through how they might cope and manage things in the small windows of time between the next steps.* (FG1)<br>– *Drilling into what somebodies plans for risk management would be at that first session so that at that results session you talk that through about where they are going to go to medically* (FG1)<br>– *I guess for me it is often who they might be going to have a chat about this sort of information with.* (FG3)<br>Drawing on intuition<br>– *There are those odd ones where I say 'you know what I am going to give your GP a call'… I guess some of it is you kind of eyeball the person and get a good hunch as to whether they are going to follow up or not.* (FG4)	**Capability:**<br>Skills working with families<br>– *skills in working with families. You know, family systems theory and how to actually work with multiple individuals in a counselling session.* (FG1)<br>Ability to develop rapport<br>– *You are going to base your level of support on the rapport you have with that patient. For some you have really excellent rapport and for some it is very abrupt. So, even though it shouldn't* be, *but it does happen, during the consultation we are inclined to support one person over the other based on the rapport that we have.* (FG2)<br>**Motivation:**<br>Beliefs about the extent to which clients should take ownership of their own healthcare<br>– *I do think they [clients] need to take some responsibility and ownership of the diagnosis or the genetic result or family communication.* (FG3)<br>– *I would say the duty of a clinical genetic counselor is discharged with something like a referral. The letter is written, it is put in the post... It is then the responsibility of the patient to come to me if nothing has happened.* (Interview 1)<br>– *You can't help people who don't want to help themselves.* (FG4)<br>Judgements of clients' ability to manage their own healthcare<br>– *When you get the re- referral from the new GP in two years and nothing has happened in between and you go and check on the system and say 'They seemed capable', but nothing has happened and you don't know why that is. But, there is no way you had time to follow up on that person who seemed like they were going to be able to do it themselves.* (FG3)<br>– *there are that certain vulnerable cohort who I am willing to go that extra mile for.* (FG4)
**Provide tailored information, support and resources:**<br>Translate, tailor and filter complex genetic information<br>– *I focus more on is what they perceive as important to them. It is all about tailoring the information to*	**Capability:**<br>Skills to understand, filter and convey relevant information and address misconceptions<br>– *We translate really complex information for people…it can be really hard to help people to understand test results and the implications of those results in a time of crisis. But I think that*

their needs. Some strategies work for some and some work for others. (FG2)<br>– *it is the ability to give them that information that they don't need to end up down a rabbit hole and help to assist them filter that information online in the future if they do need to find something else.* (FG3)<br>Provide accessible and relevant information<br>– *I provide them with information, both verbal and written information* (FG4)<br>Provide access to relevant support<br>– *Implementing or suggesting a network, who they can speak to, is there someone professional we would recommend they can speak to.* (FG4)<br>– *Not only providing support ourselves but making sure they are linked in with appropriate support structures out there to facilitate them doing the things we are expecting or hoping that they will do.* (FG1)<br>– *linking them in with a health professional who can offer more continued support moving forward.* (FG2)<br>– *breaking down the barriers of access, so where are they located ..., ok you have Greek relatives, here is the international clinic.* (FG1)	*is one of the things that GCs do really well - is that translation of really complex genetic information* (FG1)<br>– *The point is to learn how to find the things and … to filter the information. And that is our training and that is what we can bring to the room that the families themselves won't necessarily have*. (FG3)<br>– *Mis-literate is something that we have only seen in the last five years or maybe a bit more. The availability of information on the internet, not all of which is reliable. And that terrifying prospect of somebody walking into a consult with a stack of printouts thinking they already know everything about we are doing and having to undo some of that is a real challenge sometimes.* (FG1)<br>– *We have had patients who are getting information for them and their family (from DTC), and it is opening up a whole ethical dilemma because they have put their data into promethease and suddenly they supposedly have a gene mutation for a cancer gene. And then we are almost having to disprove it.* (FG1)<br>**Opportunity:**<br>Local knowledge and connections<br>– *I have a big, long list of telephone numbers for helpful people in my region* (FG3)<br>– *There is the transport sector or there is this type of funding for people coming from far away. Knowing that type of information or how to get a family in touch with the fuel rebate so they can get themselves to appointments* (FG3)<br>**Motivation:**<br>Belief in the genetic counselor role as care coordinator<br>– *It is that huge spectrum of responsibility that a genetic counselor assumes, literally from what button to press in the elevator and how is this going to affect your existential, you know, existence in the world and how are you going to assimilate this* (FG3).
**Enable behavior change:**<br>Promote collaborative decision-making and planning<br>– *Coming to a common you know, mutual plan. Not so much an agenda but a plan of contact and follow up. If they are oscillating 'how about I give you a call in another month' type of thing.* (FG1)<br>– *It is happy medium between supporting the patient to make a decision that is line with their*	**Capability:**<br>Prevention and early detection of relatives' risk<br>– *In terms of family communication and risk management genetic counselors do have training with that and we are quite good at helping patients with that, within our scope of practice.* (FG2)<br>Skills and training in behaviour change

values whilst being very aware of what is recommended and what is best practice. (FG2)<br>Activate screening/health appointment<br>− *whether people go ahead with prenatal testing or PGD/IVF and you know making the appropriate referrals* (FG2)<br>Encourage self-management/ health behavior change<br>− *The more you are kind of black and white and pushy about it, the less likely they are going to see it as a positive change or that something they want to do. So, providing them with time or exploring those barriers in such a way it is simply just an exploration* (FG2)<br>− *I have actually said to [people] 'So your response looks to me like that is not something that is important to you and that is not going to change your behavior and actually really challenge them and talk to them about why that it, is that not a priority for them.* (FG4)<br>− *set deadlines and say, so by this time next week can you let me know, or within this timeframe we should have an outcome or a result for you. (Interview 1)*<br>− *I might say 'I am going to do A, B, C, why don't you tell me a little bit about what you are going to do after you see me. Engaging them in a session, allowing them to think for themselves what they need to do.* (FG4)<br>Helping clients identify which relatives to share information with<br>− *it is very different depending on the condition, depending on the family. I think you get a really good idea on things like family communication when you take the family history. Or you talk about any aspect of 'Who you are close with, who do you share information with'.* (FG4)<br>Helping clients identify and plan what information to share with relatives<br>− *With a client we might actually get them to mentally move into the idea, and sometimes even practice the words they might use or get them to think about how they are going to do it. When might be the best time.* (FG1)<br>− *Constructing a family story that will be feasible across all of the other siblings still respecting the first person's privacy about the information.* (FG1)<br>Negotiating what can be said to whom and how the information will be shared	− *it's really skills and training. But I don't really feel like I have the skills to enact behaviour change. Maybe that's something my you just develop as you become more experienced. I don't know. But it's certainly something that I don't think I'd be confident being asked to do at the moment. (*FG5)<br>− *There are people who are open to shifting, changing, thinking, creating – you know like in a creative way, new ways of doing what we do or having different perspectives. … if the genetic counselor can't do it themselves, then obviously they're not going to be able promote the client (Interview 1)*<br>**Motivation:**<br>Beliefs about whether behavior change is directive or not<br>− *You don't want to be too pushy* (FG5)<br>− *I think one kind of, like catch, is the premise of us being non-directive, and us being non-directive, if exploring, any barriers to facilitating information sharing or screening, that it was one that was gentle and not pushy. But that that, and that is still applicable to health behavior change but, being non-directive is a little bit at odds with behavior change.* (FG2)<br>Beliefs about whether genetic counseling should be directive or non-directive<br>− *You want to be non-directive as you don't want to push people to say, go and have risk-reducing surgery… But I think there are certain areas where, I guess, pulling back on it a little bit, on the non-directiveness is appropriate, particularly if they are not having any screening whatsoever, to try and provide incentives for that.* (FG2)<br>− *I personally think non-directiveness is kind of bullshit and doesn't actually align with what we do.* (FG5)<br>− *I think people come to see us as the experts in genetics and genetic testing. To then sort of sit there and be non-directive, about particular scenarios, it doesn't gel quite well anymore. I think the non-directiveness is from before, and now, with the mainstreaming we have to be much more directive.* (FG5)<br>Beliefs about whether behavior change is within genetic counselors' scope of practice<br>− *it's an integral part of our work or at least, if you're clinical facing role, or it should be, even if it isn't now.* (Interview 1)

– *I almost always ask specifically, who do you think you are going to tell, what do you think that is going ok be like. Have you thought about how you are going to say it* (FG3)<br>– *Negotiating who we can correspond with, what information we can speak to if another relative calls us, so we are clear and they are clear with that.* (FG1)<br>Equip clients to share information with other agencies<br>– *Sometimes we write a general information letter that someone can take to teachers or depending on what the issue is we will role play how you might raise something or talk about it* (FG3)<br>Equip clients with the skills or resources to share information<br>– *Providing them the tools to do that in a way they are comfortable. Giving them the words or it could be giving them the anonymous family letter that they can then pass on in some way.* (FG3)<br>– *Talking to families about incorporating the genetic story at an age-appropriate stage.* (FG1)<br>– *We might get them to imagine how they could make contact with a person that they haven't had any contact with for a long time. Or could they use an intermediary or pass the* information on via another relative (??)<br>– *If I have already incorporated skills like 'teach back' within the setting to gage how much they have understood and what they can tell back to me.* (FG1)<br>– *A lot of the time, there will be sort of role play in the session or ok so 'who are the people in the family you might find hardest to talk to' things like that* (FG3)	– *Given that we work within genetic services that provide genetic testing to people where the subsequent recommendations are to do X, Y, and Z in certain circumstances, do we then have a moral responsibility to support or have the adequate resources, downstream resources, to suppose that behavior change?* (FG5)<br>– *I think that it's too big for us to be - just a whole - it's a whole separate, like, skillset, and - if we then think about psychologists, for example, if those are specialising in CBT, for example, it's like their whole session is based around this very issue. It's like - it's bigger than what we're able to do. We struggle with resources as it is. It's bigger than - and what we're capable of doing.* (FG5)
**Monitor behavior change:**<br>Follow up<br>– *in the cancer setting, where they will get a survey with quick things of, you know, 'who have you told, what screening have you had.. or what risk management options have you taken up* (FG2)<br>– *If we have asked them to get blood tests, or if they are going to have to do something sometimes I get them to call me when they have done it, just so you know we can chase up the results for your or touch base around then.* (FG3)<br>– *Monitoring the outcome of family communication … a place where everybody could have a secure place to put their family information in, the genetic*	**Opportunity:**<br>Time, capacity and resources<br>– *Sometimes a barrier to follow up is our limited time. Our giant workload and all of the things on our to do list at the start of each day that we know we are never going to get to all of them.* (FG3)<br>– *Some services you just don't have the resources to do that. I do know there are other places that follow them up annually but others you just cannot do that. So, I do find that actually making sure people are doing the right screening and seeing the right specialist can be quite difficult* (FG2).

test result could be hosted there, a clinic letter to relatives could be there (FG1)	– *often it's a single – one or two sessions at max, and that's it, discharged, and there's no further contact (Interview 1)*<br>Systems for appointments and follow up<br>– *it is hard when something has been recommended for them but they can't access it, so there is an 18 month wait … There is nothing you can do about it because the other department doesn't deem them as a priority* (FG3)<br>– *We don't have any structural follow up or high risk clinics to fall back on or anything like that. So, any follow up we do, has to be self-directed by us.* (FG3)
